# Supplementary material for: Factors associated with visual outcomes after cataract surgery: A cross-sectional or retrospective study in Liberia
Source: PLoS One. 2020 May 18;15(5):e0233118. doi: 10.1371/journal.pone.0233118 (PMC7233528; doi:10.1371/journal.pone.0233118)
Supplement: Attachment — Submitted filename: PONE-D-19-34050_reviewer (1).pdf [file pone.0233118.s002.pdf]

PLOS ONE

Cataract Surgery Visual Outcomes and Associated Risk Factors in Liberia

--Manuscript Draft--

**Manuscript Number:**	PONE-D-19-34050
**Article Type:**	Research Article
**Full Title:**	Cataract Surgery Visual Outcomes and Associated Risk Factors in Liberia
**Short Title:**	Visual outcomes of cataract surgery in Liberia
**Corresponding Author:**	Rohit C. Khanna, MD, MPH<br>LV Prasad Eye Institute<br>Hyderabad, INDIA
**Keywords:**	Cataract surgery; Liberia; visual acuity; visual outcome
**Abstract:**	Objective: To report the initial outcomes and associated risk factors of cataract surgery performed in Liberia<br>Methods and Analysis: Since July 2017, LV Prasad Eye Institute (LVPEI), Hyderabad, started providing eye care in Liberia. Electronic Medical Records of 573 patients operated for age-related cataract from July 2017 to January 2019 were reviewed. One eye per patient was included for analysis. All patients underwent either phacoemulsification or manual small incision cataract surgery (MSICS). Pre and postoperative uncorrected visual acuity (UCVA) and best-corrected visual acuity (BCVA) were recorded at one day, 1-3 weeks and 4-11 weeks. Main outcome measure was BCVA at 4-11 weeks; Intraoperative complications and preoperative ocular comorbidities (POC) were noted. BCVA less than 6/12 was classified as visual impairment (VI). Risk factor for VI was analysed using the logistic regression model.<br>Results: Of the 573 patients, 288 were males and 285 were females (49.7%). Mean age was 65.9±10.9 years; 14.3% had POC. The surgical technique was mainly MSICS (94.59%, n=542). At 4–11 weeks, good outcome of 6/12 or better was noted in 38.55% (UCVA) and 82.54% (BCVA). Visual acuity (VA) of 6/18 or better as UCVA and BCVA was noted in 63.5% and 88% eyes respectively. Poor outcome of less than 6/60 was noted as UCVA (11.11%) and BCVA (5.22%). Multivariable analysis showed poor visual outcomes significantly higher in patients with POC (odds ratio 3.28; 95% CI: 1.70, 6.34).<br>Conclusion: The cataract surgical outcomes in Liberia were similar to the recommended by World Health Organization; with ocular comorbidities as the only risk factor.
**Order of Authors:**	Rohit C. Khanna, MD, MPH
	Varsha M Rathi
	Edward Guizie
	Gurcharan Singh
	Kumar Nishant
	Smrita Sandhu
	Rajashekar Varda
	Anthony Vipin Das
	Gullapalli Nageswara Rao
**Additional Information:**	
**Question**	**Response**
**Financial Disclosure**<br><br>Enter a financial disclosure statement that describes the sources of funding for the	The funders had no role in study design, data collection and analysis, decision to publish, or preparation of the manuscript.

work included in this submission. Review the submission guidelines for detailed requirements. View published research articles from *PLOS ONE* for specific examples.<br><br>This statement is required for submission and **will appear in the published article** if the submission is accepted. Please make sure it is accurate.<br><br>**Unfunded studies**<br>Enter: *The author(s) received no specific funding for this work.*<br><br>**Funded studies**<br>Enter a statement with the following details:<br>• Initials of the authors who received each award<br>• Grant numbers awarded to each author<br>• The full name of each funder<br>• URL of each funder website<br>• Did the sponsors or funders play any role in the study design, data collection and analysis, decision to publish, or preparation of the manuscript?<br>• **NO** - Include this sentence at the end of your statement: *The funders had no role in study design, data collection and analysis, decision to publish, or preparation of the manuscript.*<br>• **YES** - Specify the role(s) played.<br><br>* typeset	
**Competing Interests**<br><br>Use the instructions below to enter a competing interest statement for this submission. On behalf of all authors, disclose any competing interests that could be perceived to bias this work—acknowledging all financial support and any other relevant financial or non-financial competing interests.<br><br>This statement **will appear in the published article** if the submission is accepted. Please make sure it is accurate. View published research articles from *PLOS ONE* for specific examples.	The authors have declared that no competing interests exist.

Powered by Editorial Manager® and ProduXion Manager® from Aries Systems Corporation

**NO authors have competing interests**<br><br>Enter: *The authors have declared that no competing interests exist.*<br><br>**Authors with competing interests**<br><br>Enter competing interest details beginning with this statement:<br><br>*I have read the journal's policy and the authors of this manuscript have the following competing interests: [insert competing interests here]*<br><br>* typeset	
**Ethics Statement**<br><br>Enter an ethics statement for this submission. This statement is required if the study involved:<br><br>• Human participants<br>• Human specimens or tissue<br>• Vertebrate animals or cephalopods<br>• Vertebrate embryos or tissues<br>• Field research<br><br>Write "N/A" if the submission does not require an ethics statement.<br><br>General guidance is provided below. Consult the submission guidelines for detailed instructions. **Make sure that all information entered here is included in the Methods section of the manuscript.**	The study was approved by the University of Liberia-Pacific Institute for Research & Evaluation Institution Review Board.<br>Protocol #18-05-104<br>Written consent was obtained from patients and the data was analyzed anonymously

Powered by Editorial Manager® and ProduXion Manager® from Aries Systems Corporation

**Format for specific study types**<br><br>**Human Subject Research (involving human participants and/or tissue)**<br>• Give the name of the institutional review board or ethics committee that approved the study<br>• Include the approval number and/or a statement indicating approval of this research<br>• Indicate the form of consent obtained (written/oral) or the reason that consent was not obtained (e.g. the data were analyzed anonymously)<br><br>**Animal Research (involving vertebrate animals, embryos or tissues)**<br>• Provide the name of the Institutional Animal Care and Use Committee (IACUC) or other relevant ethics board that reviewed the study protocol, and indicate whether they approved this research or granted a formal waiver of ethical approval<br>• Include an approval number if one was obtained<br>• If the study involved *non-human primates*, add *additional details* about animal welfare and steps taken to ameliorate suffering<br>• If anesthesia, euthanasia, or any kind of animal sacrifice is part of the study, include briefly which substances and/or methods were applied<br><br>**Field Research**<br><br>Include the following details if this study involves the collection of plant, animal, or other materials from a natural setting:<br>• Field permit number<br>• Name of the institution or relevant body that granted permission	
**Data Availability**<br><br>Authors are required to make all data underlying the findings described fully available, without restriction, and from the time of publication. PLOS allows rare exceptions to address legal and ethical concerns. See the PLOS Data Policy and FAQ for detailed information.	No - some restrictions will apply

Powered by Editorial Manager® and ProduXion Manager® from Aries Systems Corporation

A Data Availability Statement describing where the data can be found is required at submission. Your answers to this question constitute the Data Availability Statement and **will be published in the article**, if accepted.<br><br>**Important:** Stating 'data available on request from the author' is not sufficient. If your data are only available upon request, select 'No' for the first question and explain your exceptional situation in the text box.<br><br>Do the authors confirm that all data underlying the findings described in their manuscript are fully available without restriction?	
**Describe where the data may be found in full sentences. If you are copying our sample text, replace any instances of XXX with the appropriate details.**<br><br>• If the data are **held or will be held in a public repository**, include URLs, accession numbers or DOIs. If this information will only be available after acceptance, indicate this by ticking the box below. For example: *All XXX files are available from the XXX database (accession number(s) XXX, XXX.)*.<br>• If the data are all contained **within the manuscript and/or Supporting Information files**, enter the following: *All relevant data are within the manuscript and its Supporting Information files.*<br>• If neither of these applies but you are able to provide **details of access elsewhere**, with or without limitations, please do so. For example:<br><br>*Data cannot be shared publicly because of [XXX]. Data are available from the XXX Institutional Data Access / Ethics Committee (contact via XXX) for researchers who meet the criteria for access to confidential data.*<br><br>*The data underlying the results presented in the study are available from (include the name of the third party*	All relevant data are within the manuscript and its Supporting Information files.

and contact information or URL).<br>• This text is appropriate if the data are owned by a third party and authors do not have permission to share the data.<br><br>* typeset	
Additional data availability information:	Tick here if the URLs/accession numbers/DOIs will be available only after acceptance of the manuscript for publication so that we can ensure their inclusion before publication.

Powered by Editorial Manager® and ProduXion Manager® from Aries Systems Corporation

Manuscript
Click here to download Manuscript Liberia_outcome_plain_text.docx

Cataract Surgery Visual Outcomes and Associated Risk Factors in Liberia

Rohit C Khanna[1,2, 3, 4]

Varsha M Rathi[1,2, 3]

Edward Guizie[1]

Gurcharan Singh[1]

Kumar Nishant [1]

Smrita Sandhu[1]

Rajashekar Varda[1,2]

Anthony Vipin Das[1,3,5]

Gullapalli Nageswara Rao[1,2,3]

1. Liberia Eye Centre, John F. Kennedy Memorial Medical Centre, Monrovia, Liberia
2. Allen Foster Community Eye Health Research Centre, Gullapalli Pratibha Rao International Centre for Advancement of Rural Eyecare, LV Prasad Eye Institute, Hyderabad, India
3. Brien Holden Eye Research Centre, LV Prasad Eye Institute, Hyderabad, India
4. School of Optometry and Vision Science, University of New South Wales, Sydney, Australia
5. Department of eyeSmart EMR & AEye, L V Prasad Eye Institute

Short title: Visual outcomes of cataract surgery in Liberia

Corresponding Author:

Varsha M Rathi

varsharathi@lvpei.org

Keywords: Cataract surgery; Liberia; visual acuity; visual outcome

// Abstract

Objective: To report the initial outcomes and associated risk factors of cataract surgery performed in Liberia

Methods and Analysis: Since July 2017, LV Prasad Eye Institute (LVPEI), Hyderabad, started providing eye care in Liberia. Electronic Medical Records of 573 patients operated for age-related cataract from July 2017 to January 2019 were reviewed. One eye per patient was included for analysis. All patients underwent either phacoemulsification or manual small incision cataract surgery (MSICS). Pre and postoperative uncorrected visual acuity (UCVA) and best-corrected visual acuity (BCVA) were recorded at one day, 1-3 weeks and 4-11 weeks. Main outcome measure was BCVA at 4-11 weeks; Intraoperative complications and preoperative ocular comorbidities (POC) were noted. BCVA less than 6/12 was classified as visual impairment (VI). Risk factor for VI was analysed using the logistic regression model.

Results: Of the 573 patients, 288 were males and 285 were females (49.7%). Mean age was 65.9±10.9 years; 14.3% had POC. The surgical technique was mainly MSICS (94.59%, n=542). At 4–11 weeks, good outcome of 6/12 or better was noted in 38.55% (UCVA) and 82.54% (BCVA). Visual acuity (VA) of 6/18 or better as UCVA and BCVA was noted in 63.5% and 88% eyes respectively. Poor outcome of less than 6/60 was noted as UCVA (11.11%) and BCVA (5.22%). Multivariable analysis showed poor visual outcomes significantly higher in patients with POC (odds ratio 3.28; 95% CI: 1.70, 6.34).

Conclusion: The cataract surgical outcomes in Liberia were similar to the recommended by World Health Organization; with ocular comorbidities as the only risk factor.

Introduction

There are 253 million people blind or visually impaired worldwide. Of these 89% live in low and middle income countries (LMIC); and 55% are women.[1] Globally, cataract is the leading cause of blindness and second leading cause of visual impairment (VI).[2] In Sub-Saharan Africa 35-45% of blindness and 25-35% for VI is caused by cataract.[2] Liberia in West Africa has a population of approximately 5 million.[3] World Health Organization (WHO) estimates that approximately 1% of Liberians (approximately 35,000 people) suffer from blindness of which 50% is due to cataract.[3] In terms of human resources, there is only one ophthalmologist for one million population on an average in the sub-Saharan region.[4] This holds true even for Liberia [4] The Cataract Surgical Rate (CSR) is one of the lowest in Liberia with 81 in 2010 and 157 in 2014.[5, 6] In terms of cataract surgical outcomes, a study by Frucht-Pery and Feldman on cataract surgery in patients with leprosy in Liberia reported that a visual acuity of 20/200 or better was achieved only in 65% of patients.[7] However, this study included only 43 eyes of 30 patients.[7] There are similar reports from other African countries with poor outcomes ranging from 10 – 40%.[8, 9]

The L V Prasad Eye Institute's (LVPEI) pyramidal model of eye care delivery has a Centre of Excellence (CoE) at the top catering to a population of 50 million population with Tertiary Centres (TC) at the next level, each for 5 million population.[10] These are linked to Secondary Centres (SC) covering 0.5-1 million population mostly in rural locations, with Vision Centres (VC) at primary level for 50,000 population, and Vision Guardians (VG) for 5,000 population. The functions at each level of the pyramid are clearly delineated and demarcated. The SCs are run by one or two ophthalmologists who are trained at a TC or COE for a year. Patients from SCs are referred to TCs or COE only for advanced care and management of complex problems.[10] The model has shown good outcomes of cataract

surgery in their rural SCs.[11] The secondary centres provide comprehensive affordable, accessible and appropriate eye care irrespective of the paying capacity of the patients, and have fully equipped surgical facilities where mainly cataract surgeries and other procedures are performed.[10]

In 2014, LVPEI launched the Liberia Eye Health Initiative (LEHI), at the John F Kennedy (JFK) Medical Centre, which is the apex centre for health care in Liberia. A formal collaborative agreement was signed between LVPEI and JFK. The Liberia Eye Centre (LEC) was formally inaugurated on 24 July 2017 in Monrovia, to provide comprehensive eye care for people of Liberia. All the staff in the LEC were trained at LVPEI. Since 2018, LVPEI also started the ophthalmology residency training program in Liberia. The purpose of this study is to evaluate outcomes of cataract surgery performed, at the LEC as well as associated risk factors for poor outcomes.

Materials and Methods

We retrospectively analysed the records of patients who underwent cataract surgery in the LEC at JFK Memorial Medical Centre, Monrovia, Liberia from July 2017 to January 2019. The study was approved by the University of Liberia-Pacific Institute for Research & Evaluation Institution Review Board. The study was conducted in accordance to the tenets of Declaration of Helsinki.

The study included all patients 40 years of age or above. Only the first operated eye was included in the analysis. Data was collected from the Electronic Medical Records (EMR). The patients underwent comprehensive eye examination, which included detailed history; uncorrected visual acuity (UCVA) and best corrected visual acuity (BCVA); intraocular

pressure measurement with Goldmann applanation tonometer; dilated lens examination to assess the lens status; and stereoscopic fundus examination with +78/90 Dioptre lens as well as indirect ophthalmoscope. Pre-existing ocular comorbidities were grouped as corneal pathologies, retinal disease, glaucoma and others (non-glaucomatous optic nerve pathologies and uveitis). The systemic comorbidities included hypertension (HT), diabetes mellitus (DM), and HIV seropositive patients. The surgeon decided on the surgical technique – either a phacoemulsification or manual small incision cataract surgery (MSICS), performed with or without intraocular lens (IOL) implantation. Anterior chamber (AC) and posterior chamber (PC) IOLs were used. The intraoperative complications (posterior capsule rupture, zonular dehiscence etc) were noted. All the surgeries were performed by three surgeons as well as other visiting faculty.

The primary outcome measure was postoperative visual acuity. The visual acuity was noted preoperatively and postoperatively on day 1, between 1-3 weeks and 4-11 weeks of surgery and analysed. Visual acuity was categorized as 6/12 or better; less than 6/12 to 6/18; less than 6/18 to 6/60 and less than 6/60. Poor outcome was defined as BCVA of less than 6/12 in the operated eye.

Statistical analysis: Statistical analysis was done using Stata 13 (Statacorp, Texas). Logistic regression model was used to evaluate the association of risk factors for poor outcomes and Fisher's Exact test was used for categorical variable. A two tailed p value of <0.05 was considered statistically significant. Risk factors for poor outcomes were analysed using univariable and multivariable regressions, based on BCVA at 4–11 weeks follow up.

Results

Between July 2017 and January 2019, a total of 739 cataract surgeries were performed. Of these, 126 were bilateral cataract surgeries (252 eyes), and their first operated eye was

included in the analysis. Those 40 patients who underwent cataract surgery at age less than 40 years were also excluded. Hence, 573 eyes of 573 patients were included in the study. Table 1 shows the baseline demographics and ocular findings of these 573 patients. The mean age of the patients was 65.9±10.9 years (ranging from 40 to 99 years); and 49.7% were females. Pre-existing ocular comorbidity was present in 82 patients (14.3%) and systemic comorbidity was noted in 20 patients (3.5%).

Table 1: Demographics and baseline characteristics of the patients who underwent cataract surgery

**Variable**		**Number of patients (Percentage)**
**Age group**	40 to 49 years	38 (6.6%)
	50 to 59 years	122 (21.3%)
	60 to 69 years	202 (35.3%)
	70 years or above	211 (36.8%)
**Gender**	Male	288 (50.3%)
	Female	285 (49.7%)
**Economic status**	Paying (Paid surgeries)	306 (53.4%)
	Non-paying (Free surgeries)	267 (46.6%)
**Eye**	Right eye	329 (57.4%)
	Left eye	244 (42.6%)
**Preoperative BCVA***	Less than 6/60	289 (50.4%)
	Less than 6/18 to 6/60	94 (16.4%)
	Less than 6/12 to 6/18	75 (13.1%)
	6/12 or better	115 (20.1%)
**Systemic comorbidities**	Diabetes Mellitus	4 (0.7%)
	Hypertension	12 (2.1%)
	HIV seropositive	4 (0.7%)
**Ocular comorbidities**	None	491 (85.7%)
	Cornea	16 (2.8%)
	Glaucoma	39 (6.8%)
	Retina	9 (1.6%)
	Others[#]	18 (3.1%)

*BCVA Best corrected visual acuity; #Nonglaucomatous optic nerve disease and Uveitis

Of the 573 eyes, the MSICS technique was used in 542 (94.6%) eyes and phacoemulsification was done in 31 (5.4%) eyes. While 562 (98.1%) eyes underwent PCIOL, 11 (1.9 %) eyes were either left aphakic or had an ACIOL. Major intraoperative complications such as posterior capsular rest and zonular dehiscence were noted in 3.3% eyes. Other complications (0.9%) such as Descemet’s membrane detachment (1eye), iridodialysis (2 eyes) and would leak (2 eyes) were also noted.

Table 2 shows the preoperative and post-operative (uncorrected and best corrected) visual acuity first day; 1 to 3 weeks and 4-11 weeks follow up. At last follow up, UCVA of 6/12 or better was seen in 38.6% patients and BCVA of 6/12 or better was seen in 82.5% patients. Similarly, UCVA of 6/18 or better was seen in 63.5% patients and BCVA of 6/18 or better was seen in 88% patients. UCVA and BCVA of less than 6/60 were seen in 11.11% and 5.22% patients respectively.

Table 2: Uncorrected and Best Corrected Visual Acuity at Different Follow-up Intervals.

	**Preoperative**		**Post-operative**					
			**Day 1**		**1-3 weeks**		**4-11 weeks**	
	**n=573 (100%)**		**n=573 (100%)**		**n= 509 (88.83%)**		**n=441 (76.96%)**	
VA	UCVA#	BCVA*	UCVA#	BCVA*	UCVA#	BCVA*	UCVA#	BCVA*
6/12 or better	44 (7.7%)	115 (20.1%)	249 (43.5%)	419 (73.1%)	224 (44.0%)	402 (79.0%)	170 (38.5%)	364 (82.5%)
Less than 6/12 to 6/18	48 (8.4%)	75 (13.1%)	150 (26.2%)	50 (8.7%)	118 (23.2%)	33 (6.5%)	110 (24.9%)	24 (5.4%)
Less than 6/18 to 6/60	132 (23.0%)	94 (16.4%)	114 (19.9%)	53 (9.2%)	109 (21.4%)	35 (6.9%)	112 (25.4%)	30 (6.8%)
Less than 6/60	349 (60.9%)	289 (50.4%)	60 (10.5%)	51 (8.9%)	58 (11.4%)	39 (7.7%)	49 (11.1%)	23 (5.2%)

#UCVA Best corrected visual acuity; *BCVA Best corrected visual acuity

At 4-11 weeks, there were 441 (77%) patients available for follow-up. Table 3 shows the demographics and ocular findings of those who reported for follow up and those who did not, between 4-11 weeks. There was no difference between those lost to follow up and those who were available for follow up, in terms of gender, paying and non-paying status, eye operated, type of surgery, use of intraocular lens, choice on intracameral antibiotics, and intraoperative complications. However, there was a difference in terms of age group, operating surgeon and presence of ocular comorbidity.

Table 3 shows the demographics and ocular findings of those who followed up and who did not follow up between 4-11 weeks of follow up.

		Available	Not available	P value
		n=441	n=132	
**Age group**	40-49 years	34 (7.7%)	4 (3.0%)	
	50-59 years	90 (20.4%)	32 (24.2%)	
	60-69 years	146 (33.1%)	56 (42.4%)	
	>=70 years	171 (38.8%)	40 (30.3%)	
				0.04
**Gender**	Male	219 (49.7%)	69 (52.3%)	
	Female	222 (50.3%)	63 (47.7%)	
				0.6
**Economic status**	Paying(Paid surgeries)	227 (51.5%)	79 (59.8%)	
	Non-paying (Free surgeries)	214 (48.5%)	53 (40.2%)	
				0.09
**Eye**	Right eye	255 (57.8%)	74 (56.1%)	
	Left eye	186 (42.2%)	58 (43.9%)	
				0.72
**Type of surgery**	Phacoemulsification	26 (5.9%)	5 (3.8%)	
	MSICS#	415 (94.1%)	127 (96.2%)	
				0.35

**Type of IOL**	PC IOL^	430 (97.5%)	132 (100.0%)	
	No IOL* or AC IOL[&]	11 (2.5%)	0 (0.0%)	
				0.07
**Intracameral antibiotics**	Cefuroxime	49 (11.1%)	10 (7.6%)	
	Moxifloxacin	334 (75.7%)	117 (88.6%)	
				0.21
**Intraoperative Complications**	No or minor complications	427 (96.8%)	127 (96.2%)	
	Major complications	14 (3.2%)	5 (3.8%)	
				0.73
**Surgeon category**	Faculty 1	140 (31.7%)	29 (22.0%)	
	Faculty 2	249 (56.5%)	53 (40.2%)	
	Faculty 3	46 (10.4%)	15 (11.4%)	
	Visiting Faculty	6 (1.4%)	35 (26.5%)	
				<0.001
**Systemic comorbidities**	Absent	425 (96.4%)	128 (97%)	
	Present	16 (3.6%)	4 (3%)	
				0.74
**Ocular comorbidities**	Absent	370 (83.9%)	121 (91.7%)	
	Present	71 (16.1%)	11 (8.3%)	
				0.03

#MSICS – Manual small incision cataract surgery; *IOL – Intraocular lens; ^PCIOL – Posterior chamber intraocular lens; [&]AC IOL – Anterior chamber intraocular lens

Table 4 shows the differences between the demographics and ocular characteristics of patients who had good versus poor outcomes. Those with poor outcomes were older (p=0.04); had higher intraoperative complications (p<0.001) as well as associated ocular comorbidity (<0.001); and had no IOL and AC IOL (p=0.001).

Table 4: Difference between demographics and ocular characteristic of good versus poor outcome

		BCVA 6/12 or better	BCVA less than 6/12	Total	P value
		n=364	n=77	n=441	
Age group	40-49 years	29 (8.0%)	5 (6.5%)	34	
	50-59 years	79 (21.7%)	11 (14.3%)	90	
	60-69 years	126 (34.6%)	20 (26.0%)	146	
	>=70 years	130 (35.7%)	41 (53.2%)	171	

					0.04
Gender	Male	178 (48.9%)	41 (53.2%)	219	
	Female	186 (51.1%)	36 (46.8%)	222	
					0.49
Economic status	Paying(Paid surgeries)	190 (52.2%)	37 (48.1%)	227	
	Non-paying (Free surgeries)	174 (47.8%)	40 (51.9%)	214	
					0.51
Eye	Right eye	207 (56.9%)	48 (62.3%)	255	
	Left eye	157 (43.1%)	29 (37.7%)	186	
					0.38
Type of surgery	Phacoemulsification	22 (6.0%)	4 (5.2%)	26	
	MSICS[#]	342 (94.0%)	73 (94.8%)	415	
					0.77
Type of IOL	PC IOL^	359 (98.6%)	71 (92.2%)		
	No IOL* or AC IOL[&]	5 (1.4%)	6 (7.8%)		
					0.001
Intracameral antibiotics	Cefuroxime	36 (9.9%)	13 (16.9%)	49	
	Moxifloxacin	281 (77.2%)	53 (68.8%)	334	
					0.07
Intraoperative complications	Absent	357 (98.1%)	70 (90.9%)	427	
	Present	7 (1.9%)	7 (9.1%)	14	
					<0.001
Surgeon category	Faculty 1	119 (32.7%)	21 (27.3%)	140	
	Faculty 2	197 (54.1%)	52 (67.5%)	249	
	Faculty 3	43 (11.8%)	3 (3.9%)	46	
	Visiting Faculty	5 (1.4%)	1 (1.3%)	6	
					0.09
Systemic comorbidities	Absent	351 (96.4%)	74 (96.1%)	425	
	Present	13 (3.6%)	3 (3.9%)	16	
					0.89
Ocular comorbidities	Absent	319 (87.6%)	51 (66.2%)	370	
	Present	45 (12.4%)	26 (33.8%)	71	
					<0.001

#MSICS – Manual small incision cataract surgery; *IOL – Intraocular lens; ^PCIOL – Posterior chamber intraocular lens; [&]AC IOL – Anterior chamber intraocular lens

Table 5 shows univariable and multivariable association of demographic and ocular surgical factors with poor outcomes (BCVA of less than 6/12 at 4–11 weeks follow-up). In univariable analysis, poor outcomes were associated with presence of ACIOL/No IOL (OR

6.07; 95% CI:1.80, 20.43); intraoperative complications (OR5.10; 95% CI: 1.73, 15.00); and associated ocular comorbidities (OR3.61; 95% CI: 2.05, 6.37). However in multivariable analysis, the only significant factor was presence of ocular comorbidities (OR3.28; 95% CI: 1.70, 6.34).

Table 5 shows univariable and multivariable association of demographics, surgical factors with visual outcome of 6/12 or better at 4–11 weeks follow-up

	**Univariable analysis**		**Multivariable analysis**	
Variable	Odds Ratio (95% CI[$])	P value	Odds Ratio (95% CI[$])	P value
**Age group**				
40-49 years	Reference		Reference	
50-59 years	0.81(0.26, 2.52)	0.71	1.16 (0.27, 4.96)	0.84
60-69 years	0.92 (0.32, 2.66)	0.88	1.29 (0.33, 5.06)	0.71
>=70 years	1.83 (0.66, 5.03)	0.24	3.02 (0.81, 11.28)	0.1
**Gender**				
Male	Reference		Reference	
Female	0.84 (0.51, 1.38)	0.49	0.81(0.45, 1.47)	0.49
**Economic status**				
Paying(Paid surgeries)	Reference		Reference	
Non-paying (Free surgeries)	1.18 (0.72, 1.93)	0.51	1.00 (0.55, 1.81)	0.99
**Eye**				
Right eye	Reference		Reference	
Left eye	0.80 (0.48, 1.32)	0.38	0.82 (0.46, 1.49)	0.52
**Type of surgery**				
Phacoemulsification	Reference		Reference	
MSICS[#]	1.17 (0.39, 3.51)	0.77	0.65 (0.19, 2.20)	0.49
**Type of IOL**				
PC IOL^	Reference		Reference	
No IOL* or AC IOL[&]	6.07 (1.80, 20.43)	0	3.97 (0.50, 31.66)	0.19
**Intracameral antibiotics**				
Cefuroxime	Reference		Reference	
Moxifloxacin	0.52 (0.26, 1.05)	0.07	0.59 (0.27, 1.30)	0.19
**Intraoperative Complications**				
Absent	Reference		Reference	
Present	5.10 (1.73, 15.00)	<0.001	4.30 (0.68, 27.01)	0.12
**Surgeon category**				
Faculty 1	Reference		Reference	
Faculty 2	1.50 (0.86, 2.61)	0.16	1.67 (0.79, 3.54)	0.18
Faculty 3	0.40 (0.11, 1.39)	0.15	0.68 (0.17, 2.73)	0.59
Visiting Faculty	1.13 (0.13, 10.19)	0.91	0.96 (0.09, 10.67)	0.97

**Systemic comorbidities**				
Absent	Reference		Reference	
Present	1.09 (0.30, 3.94)	0.89	0.93 (0.23, 3.81)	0.92
**Ocular comorbidities**				
Absent	Reference		Reference	
Present	3.61 (2.05, 6.37)	<0.001	3.28 (1.70, 6.34)	<0.001

#MSICS – Manual small incision cataract surgery; *IOL – Intraocular lens; ^PCIOL – Posterior chamber intraocular lens; [&]AC IOL – Anterior chamber intraocular lens; [$]CI – Confidence interval

Discussion

One of the indicators for quality of cataract surgery is the outcome of surgery. Hence, periodic monitoring of outcomes should be an essential component of any hospital quality audit. We reported here the outcomes of cataract surgeries performed by in a new facility built in Liberia. There is very little data on outcomes of cataract surgery from Liberia, due to limited number of surgeries. We started this new facility in July 2017, and installed the Electronic Medical Records (EMR) system to collect and store patient data on a daily basis. This is a good source for monitoring the outcomes of cataract surgery.

The only report from Liberia by Frucht-Pery and Feldman, on cataract surgical outcomes in patients with leprosy, showed that a visual acuity of 20/200 or better was achieved in 65% of patients.[7] However, this study published more than 25 years ago, included only 43 eyes and the surgical technique used was intracapsular or extracapsular cataract extraction.[7] In our setting, a majority of the surgeries were MSICS with IOL implantation, and had better outcomes. At last follow up, UCVA of 6/12 or better was seen in 38.6% patients and BCVA of 6/12 or better was seen in 82.5% patients. Similarly, UCVA of 6/18 or better was seen in 63.5% patients and BCVA of 6/18 or better was seen in 88% patients. Similar outcomes were reported in many other countries in Asia and India.[12-15] MSICS was the primary surgical technique in all these studies also.[12-15] There are very few reports on outcomes of cataract

surgery from Africa, and outcomes reported by these are not encouraging, except a few centres.[16, 17] One of the reasons for this was the availability of well-trained surgeons performing high volume work in these centres. However, outcomes were better than many other studies from Africa, including PRECOG study.[18-21] This could be due to availability of well-trained surgeons, accurate biometry in all cases, availability of equipment and consumables, and well trained staff.[22] Another possible reason for poor outcomes reported in Africa could be because the earlier outcome studies in Africa were based on extracapsular cataract extraction (ECCE). In our study, surgeries were performed using MSICS technique. The outcomes of MSICS have been reported to be better than ECCE.[12-15, 18] The outcomes were also similar to the recommended rates of WHO for BCVA (BCVA of 90% having 6/18 or better), but were less than what is recommended for UCVA.[23] There were a significant number of patients blind or with severe visual impairment (SVI) (VA less than 6/60) preoperatively; whereas only 5% had VA of less than 6/60 postoperatively at 4-11 weeks. This implies better quality of life for those blind or SVI before cataract surgery. This also suggests that there are a significant number of blind and SVI in the population who need intervention on a priority basis.

Major intraoperative complications were found in 3.3% eyes, and these are within acceptable standard stated by WHO (less than 5%). These were mainly due to posterior capsular rent or zonular dehiscence. This was slightly higher than other studies from India and Nepal [12-15] but less than most studies from Africa.[19-21] Mavrakanas et al reported an overall complication rate of 8.2% with less in MSICS surgeries (5.3%) as compared to ECCE (10.2%).[19] However, these surgeries were carried out by cataract surgeons undergoing training.[19] Complication rate was also much lower than what was reported on PRECOG and other studies.[21] However, complications were higher compared to Matta et al (1.4%)

and Sherwin et al (2.5%).[11, 24] Unlike other studies, we did not find age or presence of a complication as a risk factor for poor outcomes.[17, 25] This could be due to lesser number of complications as well as adequate management of these complications.[25] No devastating complications like expulsive choroidal haemorrhage or endophthalmitis were report, though the number of surgeries was less.

In univariable analysis, poor outcomes were associated with presence of ACIOL/No IOL (OR 6.07; 95% CI:1.80, 20.43), intraoperative complications (OR 5.10; 95% CI: 1.73, 15.00), and associated ocular comorbidities (OR 3.61; 95% CI: 2.05, 6.37). However in multivariable analysis, the only significant factor was presence of ocular comorbidities (OR3.28; 95% CI: 1.70, 6.34). This was similar to the reports from other studies.[12, 17, 18] This suggests that a good preoperative comprehensive eye examination by a trained ophthalmologist would be necessary to identify ocular comorbidities that are likely to affect outcomes. Patients with ocular comorbidities, can then be counselled with a clear explanation on the outcomes of surgery.

One of the limitations of the study could be the patients who were lost to last follow up (132 patients, 23%). However, when we compared those available and those lost to follow up, we found that they differed in terms of age group, operating surgeon, and presence of ocular comorbidity. Those available for follow up had higher prevalence of ocular comorbidity. Hence, this could underestimate the good outcomes at last follow up. PRECOG study also showed good correlation between early outcome (3 or fewer days) and 40 days or more post operatively. It also showed that eyes with borderline or poor outcome at discharge tend to improve and achieve better vision at 4-6 months. Hence, the results obtained from those available for follow up can underestimate the good outcomes.

In conclusion, this is the first study to report on the outcomes of cataract surgeries in Liberia. Permanent infrastructure, uniform protocols, and well-trained staff in all cadres can improve uptake of cataract surgeries in future. Regular monitoring and follow-up will improve the outcomes further. A Residency program has been started in Liberia recently and this article is one of the first articles from Liberia and outcomes of residents will be compared and reported in future.

References

1. Bourne RRA, Flaxman SR, Braithwaite T, Cicinelli MV, Das A, Jonas JB, et al. Magnitude, temporal trends, and projections of the global prevalence of blindness and distance and near vision impairment: a systematic review and meta-analysis. The Lancet Global health. 2017;5(9):e888-e97. doi: 10.1016/S2214-109X(17)30293-0.
2. Flaxman SR, Bourne RRA, Resnikoff S, Ackland P, Braithwaite T, Cicinelli MV, et al. Global causes of blindness and distance vision impairment 1990-2020: a systematic review and meta-analysis. The Lancet Global health. 2017;5(12):e1221-e34. doi: 10.1016/S2214-109X(17)30393-5.
3. https://www.seeintl.org/countries/liberia/. Last accessed 5th August 2019
4. Resnikoff S, Felch W, Gauthier TM, Spivey B. The number of ophthalmologists in practice and training worldwide: a growing gap despite more than 200,000 practitioners. The British journal of ophthalmology. 2012;96(6):783-7. doi: 10.1136/bjophthalmol-2011-301378.
5. Cataract surgical rates. Community eye health. 2017;30(100):88-9.
6. Wang W, Yan W, Fotis K, Prasad NM, Lansingh VC, Taylor HR, et al. Cataract Surgical Rate and Socioeconomics: A Global Study. Investigative ophthalmology & visual science. 2016;57(14):5872-81. doi: 10.1167/iovs.16-19894.
7. Frucht-Pery J, Feldman ST. Cataract surgery in a leprosy population in Liberia. International journal of leprosy and other mycobacterial diseases : official organ of the International Leprosy Association. 1993;61(1):20-4.
8. Oladigbolu KK, Rafindadi AL, Mahmud-Ajeigbe AF, Chinda D, Pam V, Samaila E. Outcome of cataract surgery in rural areas of Kaduna State, Nigeria. Annals of African medicine. 2014;13(1):25-9. doi: 10.4103/1596-3519.126943.

9. Rabiu MM, Kyari F, Ezelum C, Elhassan E, Sanda S, Murthy GV, et al. Review of the publications of the Nigeria national blindness survey: methodology, prevalence, causes of blindness and visual impairment and outcome of cataract surgery. Annals of African medicine. 2012;11(3):125-30. doi: 10.4103/1596-3519.96859.
10. Rao GN. The Barrie Jones Lecture-Eye care for the neglected population: challenges and solutions. Eye. 2015;29(1):30-45. doi: 10.1038/eye.2014.239.
11. Matta S, Park J, Palamaner Subash Shantha G, Khanna RC, Rao GN. Cataract Surgery Visual Outcomes and Associated Risk Factors in Secondary Level Eye Care Centers of L V Prasad Eye Institute, India. PloS one. 2016;11(1):e0144853. doi: 10.1371/journal.pone.0144853.
12. Gogate P, Vakil V, Khandekar R, Deshpande M, Limburg H. Monitoring and modernization to improve visual outcomes of cataract surgery in a community eyecare center in western India. Journal of cataract and refractive surgery. 2011;37(2):328-34. doi: 10.1016/j.jcrs.2010.08.034.
13. Gogate PM, Deshpande M, Wormald RP, Deshpande R, Kulkarni SR. Extracapsular cataract surgery compared with manual small incision cataract surgery in community eye care setting in western India: a randomised controlled trial. The British journal of ophthalmology. 2003;87(6):667-72. doi: 10.1136/bjo.87.6.667.
14. Hennig A, Kumar J, Yorston D, Foster A. Sutureless cataract surgery with nucleus extraction: outcome of a prospective study in Nepal. The British journal of ophthalmology. 2003;87(3):266-70. doi: 10.1136/bjo.87.3.266.
15. Venkatesh R, Muralikrishnan R, Balent LC, Prakash SK, Prajna NV. Outcomes of high volume cataract surgeries in a developing country. The British journal of ophthalmology. 2005;89(9):1079-83. doi: 10.1136/bjo.2004.063479.

16. Limburg H, Foster A, Gilbert C, Johnson GJ, Kyndt M, Myatt M. Routine monitoring of visual outcome of cataract surgery. Part 2: Results from eight study centres. The British journal of ophthalmology. 2005;89(1):50-2. doi: 10.1136/bjo.2004.045369.
17. Yorston D, Gichuhi S, Wood M, Foster A. Does prospective monitoring improve cataract surgery outcomes in Africa? The British journal of ophthalmology. 2002;86(5):543-7. doi: 10.1136/bjo.86.5.543.
18. Lindfield R, Kuper H, Polack S, Eusebio C, Mathenge W, Wadud Z, et al. Outcome of cataract surgery at one year in Kenya, the Philippines and Bangladesh. The British journal of ophthalmology. 2009;93(7):875-80. doi: 10.1136/bjo.2008.152744.
19. Mavrakanas N, Dhalla KA, Jecha J, Kapesa I, Odouard C, Murdoch I. Results and safety profile of trainee cataract surgeons in a community setting in East Africa. Indian journal of ophthalmology. 2016;64(11):818-21. doi: 10.4103/0301-4738.195594.
20. Olawoye OO, Ashaye AO, Bekibele CO, Ajayi BG. Visual outcome after cataract surgery at the university college hospital, ibadan. Annals of Ibadan postgraduate medicine. 2011;9(1):8-13. doi: 10.4314/aipm.v9i1.72428.
21. Congdon N, Yan X, Lansingh V, Sisay A, Muller A, Chan V, et al. Assessment of cataract surgical outcomes in settings where follow-up is poor: PRECOG, a multicentre observational study. The Lancet Global health. 2013;1(1):e37-45. doi: 10.1016/S2214-109X(13)70003-2.
22. Buchan JC, Dean WH, Foster A, Burton MJ. What are the priorities for improving cataract surgical outcomes in Africa? Results of a Delphi exercise. International ophthalmology. 2018;38(4):1409-14. doi: 10.1007/s10792-017-0599-y.

23. Limburg H, Foster A, Gilbert C, Johnson GJ, Kyndt M. Routine monitoring of visual outcome of cataract surgery. Part 1: Development of an instrument. The British journal of ophthalmology. 2005;89(1):45-9. doi: 10.1136/bjo.2004.045351.
24. Sherwin JC, Dean WH, Schaefers I, Courtright P, Metcalfe N. Outcomes of manual small-incision cataract surgery using standard 22 dioptre intraocular lenses at Nkhoma Eye Hospital, Malawi. International ophthalmology. 2012;32(4):341-7. doi: 10.1007/s10792-012-9565-x.
25. Fortane M, Bensaid P, Resnikoff S, Seini K, Landreau N, Paugam JM, et al. Outcomes of cataract surgery performed by non-physician cataract surgeons in remote North Cameroon. The British journal of ophthalmology. 2019;103(8):1042-7. doi: 10.1136/bjophthalmol-2018-312428.
