# Supplementary material for: Factors associated with visual outcomes after cataract surgery: A cross-sectional or retrospective study in Liberia
Source: PLoS One. 2020 May 18;15(5):e0233118. doi: 10.1371/journal.pone.0233118 (PMC7233528; doi:10.1371/journal.pone.0233118)
Supplement: Attachment — Submitted filename: PONE-_Prof. Khanna.docx [file pone.0233118.s003.docx]

PONE-D-19-34050: Cataract Surgery Visual Outcomes and Associated Risk Factors in Liberia

By Professor Khanna et al.

Dear Professor Khanna,

Thank you for submitting the manuscript in PLOS One. Sorry for being late to provide feedback on time. This is a matter non-response from the reviewers.

I would consider the manuscript needs significant improvement.

Should not the title be more reflective if it was: Factors associated with Visual Outcomes after Cataract Surgery: A cross-sectional or retrospective study in Liberia

Abstract: The objective seems to be good as a Title.

Conclusion: Conclusions do not support the results. How do we know if these are similar to the WHO outcomes? Either it could be said “very high or give the data from WHO”.

Results: Lines 119-122 should go under Materials and Methods. Also, same information is repeated twice.

Table 2: While UCVA is better than 6/12 for 44 for UCVA 115 (20%), did they need any surgery? If they were referred in future, could have been saved resources and extend the facilities who need urgent treatment. Please explain the rationale behind it.

Table is very difficult to understand its meaning. How the outcome sustains in different VA categories would be appropriate. For example, of 115 (20%) had VA 6/12 at baseline compared to 82.5% after week 4. This is a fascinating improvement.

Then, 289 (50.4%) had worse than 6/60 at baseline compared to 5.2% at week 4. This means, approximately 289-23= 266 improved their vision from 6/60, i.e. 92% got improvement. Is it correct? In case of 6/12 or better, 364-115 = 249 i.e., 68% got improvement.

Worse the VA, more the improvement after surgery. Is it not the message?

Table 2: Please remove the % to fit and make the Table better.

Surgeon category: Did Faculty 2 performed worse? Is there any explanation of this significant finding?

Please combine Table 4 and 5 together. When the univariate analysis shows non-significant association, no need to go for multi-variate adjustment. In fact, all are non-significant except the last one which could be presented in the text.

Conclusion:

“In conclusion, this is the first study to report on the outcomes of cataract surgeries in Liberia. This is not a conclusion. It could be said “this first study conducted in Liberia reported……………….”.

Permanent infrastructure, uniform protocols, and well-trained staff in all cadres can improve uptake of cataract surgeries in future. It is not clear with which results this conclusion is linked.

Regular monitoring and follow-up will improve the outcomes further.

A Residency program has been started in Liberia recently and this article is one of the first articles from Liberia and outcomes of residents will be compared and reported in future. Is it a conclusion or strength? Alternatively, are we interest to see what we have instead of potential outcome?

I would consider the conclusion should be better reflected of the findings.
